# Case Report: Durable remission in stage IV melanoma following strategic immune checkpoint inhibitor class rotation and rechallenge

**DOI:** 10.3389/fonc.2026.1875467

**Published:** 2026-07-16

**Authors:** Jamila Raja, Shuo Li, Asal Patterson, Steve R. Martinez, Perry A. Soriano, Xiaowen Wang, Peter Y. Z. Jiang

**Affiliations:** 1 Washington State University Elson S Floyd College of Medicine Internal Medicine Residency, Everett, WA, United States; 2 Department of Surgical Oncology, Washington State University Elson S Floyd College of Medicine, Everett, WA, United States; 3 Department of Hematology and Oncology, Optum-Washington (WA), and Providence Regional Cancer Partnership & Medical Center-Everett, Washington State University Elson S Floyd College of Medicine, Everett, WA, United States

**Keywords:** case report, immune checkpoint inhibitor, immune related adverse events, immunotherapy, melanoma

## Abstract

Immune checkpoint inhibitors (ICIs) have significantly improved outcomes in patients with regional and distant melanoma metastases. Severe immune-related adverse events (irAEs) occur in 20% (single agent) to nearly 50% (dual agents) of patients and may necessitate treatment discontinuation. Upon disease progression, the safety and efficacy of ICI rechallenge remains uncertain. We report a 59-year-old male with an advanced nodular melanoma who initially received Nivolumab therapy but developed grade 4 irAEs requiring treatment cessation. Following appropriate treatment and resolution of the irAEs, rotation to an ICI agent targeting a different pathway, Ipilimumab, was trialed followed by rechallenge with Nivolumab. These sequential treatment strategies resulted in complete remission without recurrence of prior irAEs. Notably, the patient has remained off therapy and disease-free for nearly five years to date. This case highlights a strategic approach to ICI rechallenge in melanoma after severe irAEs. This approach leveraged the complementary mechanisms of CTLA-4 blockade, which mediates T-cell priming and expansion of the effector cells, and PD-1 blockade, which restores exhausted T-cell function. Rotation of ICI agents may have allowed reorganization of the immune repertoire before re-exposure to PD-1 blockade. Our findings provide further evidence that, in selected patients, ICI rechallenge after significant toxicity can provide meaningful antitumor activity without recurrence of prior irAEs.

## Graphical Abstract

**Figure:**
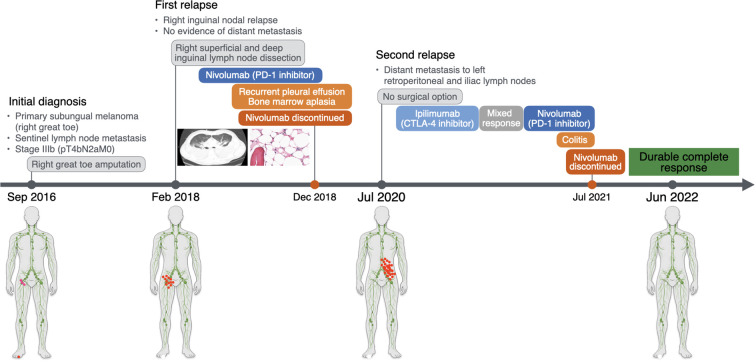
Clinical timeline of melanoma recurrence and sequential immune checkpoint inhibitor therapy. Schematic overview of the patient’s disease course, including initial diagnosis, nodal and metastatic recurrence, sequential immune checkpoint inhibitor therapy, treatment-related adverse events, and durable complete remission following nivolumab rechallenge.

## Introduction

Immune checkpoint inhibitors (ICIs) have transformed the treatment of advanced (American Joint Committee on Cancer, AJCC, Stage III-IV) melanoma. Severe immune-related adverse events (irAEs) are common and may complicate treatment necessitating discontinuation of therapy. In such circumstances, rechallenge with agents targeting alternative pathways may reduce the risk of recurrent toxicity while maintaining antitumor efficacy. Management of irAEs typically requires immunosuppressive therapy and discontinuation of the offending ICI. When disease control has not been achieved or subsequent progression occurs, it can present a therapeutic dilemma. Rechallenge with immunotherapy after severe toxicity remains controversial with limited guiding evidence. Nonetheless, in select patients, rechallenge may be both feasible and effective. This complex clinical scenario highlights the importance of individualized risk–benefit assessment when considering resumption of ICI.

We present a case of advanced nodular melanoma complicated by severe immune-mediated trilineage bone marrow aplasia during PD-1 inhibitor therapy. Following immunotherapy discontinuation and appropriate management of irAEs, the patient underwent rechallenge with alternative ICI and achieved complete remission. The patient has remained disease-free and off active therapy for 4.5 years. This case highlights the evolving management strategies in melanoma, the potential for safe reintroduction of immunotherapy after severe toxicity, and the importance of balancing efficacy with safety.

## Case description

A 59-year-old male presented with recurrent bleeding and nail loss of the right great toe, which led to the diagnosis of nodular melanoma. He underwent right great toe amputation and sentinel lymph node biopsy. The final AJCC stage was IIIB (pT4bN2aM0) without BRAF mutation. A year-long course of adjuvant immunotherapy was recommended but declined by the patient due to social circumstances. He went onto active surveillance.

18 months from his initial diagnosis, symptomatic right inguinal adenopathy developed. Right superficial and deep inguinal lymph node dissection revealed 2 of 13 nodes positive for recurrent melanoma with extra-nodal extension. Imaging with brain MRI and whole-body PET/CT did not reveal distant metastasis. Nivolumab 240mg given intravenously every 2 weeks was initiated with a planned 1-year course. After 14 doses or 7 months, treatment was discontinued due to severe irAEs, including Grade 2 recurrent pleural effusion and Grade 4 bone marrow trilineage aplasia (CBC with ANC 0.6, Hgb 12.5g, Platelets 8k and bone marrow biopsy with bone marrow cellularity 20-30%). Work-up with bone marrow biopsy investigating malignancy as the etiology for the irAEs was negative. The patient was given two courses of prednisone 60mg for aplasia, the first over 15 days and the second with a long taper over two months. He was also given one course of prednisone 80mg with taper over two months for his pleural effusions, all resulting in a full recovery.

Surveillance imaging continued periodically.

18 months from the last dose of Nivolumab, newly enlarged adenopathy involving the left retroperitoneal and iliac lymph nodes developed. CT-guided core needle biopsy confirmed recurrent melanoma. Molecular sequencing revealed a negative BRAF V660E mutation, but a positive KRAS mutation.

Given prior severe irAEs with an ICI agent targeting the PD1 pathway, an alternative agent targeting the CTLA4 pathway was selected. A strategic Ipilimumab ramp-up dose was initiated starting with 1mg/kg for one dose, then 2mg/kg for one dose, then after favorable tolerability, the dose was escalated to 3 mg/kg. Each dose was three weeks apart for a total of four cycles without irAEs. Radiographic evaluation at the conclusion of therapy demonstrated a mixed response. Short interval re-imaging revealed persistent adenopathy, but no new disease sites developed. Extensive counseling and discussion regarding rotation of ICI agents and possible Nivolumab rechallenge was conducted. The patient consented four months after the last dose of Ipilimumab.

Rechallenge with Nivolumab 480mg every four weeks resulted in further disease control and tumor size reduction until a complete response was achieved. After 4 months of therapy, however, Nivolumab was again discontinued due to the development of irAE with Grade 2 colitis, but no recurrence of prior bone marrow aplasia or pleural effusion. The colitis was initially treated with prednisone 60mg over six weeks, but symptoms recurred so he was treated with prednisone 60mg and two doses of infliximab over six weeks. No further recurrence of colitis was seen after the second course.

As of the date of this manuscript writing (June 2026), the patient’s melanoma remains in complete radiographic remission at 5 years after the last dose of Nivolumab and without sequalae of prior irAEs.

## Discussion

This case demonstrates the evolution of managing melanoma with ICIs and the application of a strategy that takes advantage of T-cell regulation. In 2016, the standard adjuvant treatment for AJCC stage IIIB melanoma was Ipilimumab, a CTLA-4 check point inhibitor, which the patient declined. After the results of the CheckMate 238 trial, first-line adjuvant treatment changed to PD1 check point inhibitor, such as Nivolumab, due to improved recurrence-free survival (1). Our patient received Nivolumab upon disease recurrence.

Unfortunately, the patient developed a rare but severe irAE: bone marrow trilineage aplasia. The pathogenesis of this bone marrow trilineage aplasia likely reflects disruption of immune tolerance resulting from PD-1 blockade unleashing autoreactive T cells that target hematopoietic progenitors, resulting in marrow failure. Similar cases of immune-mediated aplastic anemia have been reported in patients treated with PD-1 inhibitors (2). Importantly, the ability of some patients to recover suggests that homeostasis can be reestablished with time and appropriate management.

When our patient relapsed and developed distant metastasis, re-induction with an alternative pathway agent, Ipilimumab, was cautiously pursued. This was followed by a re-challenge with Nivolumab, after the patient demonstrated good tolerance. These decisions rest on CTLA-4 inhibition acting earlier than PD-1 blockade in the immune response and enhancing T-cell priming and activation (3).

The addition of Ipilimumab provided several potential benefits. First, it achieved disease control for approximately eight months, allowing a prolonged interval from prior ICI exposure and the associated irAE. This interval may have permitted recovery of immune homeostasis and reorganization of the T-cell receptor repertoire by reducing the pool of pathogenic autoreactive clones, potentially reducing the risk of recurrent irAEs upon subsequent rechallenge. In addition, CTLA-4 blockade may have facilitated the recruitment and expansion of novel anti-melanoma T-cell clones, thereby enhancing antitumor immune activity.

The favorable outcome in this case may be attributable to the patient’s younger age, nodal-only metastasis, and good performance status; however, leveraging the distinct mechanisms of CTLA-4 and PD-1 pathway blockade should not be discounted. CTLA-4 pathway blockade is known to enhance initial T-cell activation and expansion of effector T cells, while PD-1 pathway blockade acts to prevent these T-cell exhaustion or apoptosis, thus maintaining and prolonging the antitumor immune response (4). The combination or sequential use of these agents boosts T cell numbers and sustains their function. Other studies have shown that patients receiving anti-CTLA-4 and anti-PD1 therapies show early peripheral T cell expansion and increased cytokines, suggesting more proinflammatory T cell phenotypes (5). Strategic checkpoint rotation with low-dose Ipilimumab to promote T-cell expansion followed by carefully timed Nivolumab reintroduction to reinforce effector function, as demonstrated in this case, may represent a valuable therapeutic approach. The rationale for this treatment sequence is also supported by data from a Dutch registry analysis where rechallenge with Ipilimumab-Nivolumab produced response rates of 36-40% with durable benefits among responders (6).

A recent study reported retreatment with PD-1 inhibitors in patients who discontinued therapy due to treatment-related toxicity resulted in disease control for a substantial subset of patients (7). While the risk of recurrence of the initial irAE or the development of a new irAE depends on the organs involved and the severity of the initial event, retreatment remains a promising option with favorable anti-tumor activity.

Rechallenge with ICIs after irAEs is likely safer than previously assumed, though the risk of recurrent toxicity is not trivial. A 2021 systematic review and meta-analysis of nearly 800 cases reported incidence of all irAE after rechallenging was about 34%, with high-grade events at around 12%. The efficacy of the rechallenge also remained comparable to the initial treatment with response rate around 43% and disease control rate at around 72% (8). Additionally, there are important caveats highlighted in a retrospective analysis with a cohort of 93 patients who resumed anti-PD1 therapy after severe irAEs. Rechallenge was associated with a substantial risk of a second irAE with over half of patients experiencing a recurrence of the same or a new toxicity (9). In a similar study of patients who discontinued combined CTLA4 and PD1 inhibitor therapy due to irAEs, 18% had recurrence of the same irAE and over 20% developed a different irAE (10).

Given the limited prospective data, retrospective experience currently guides rechallenge strategies for ICI. “Class switching” strategies can be utilized to leverage the distinct mechanisms between CTLA4 and PD1 agents, potentially reducing the risk of producing the same toxicity. Our patient’s sustained complete remission following CTLA-4 re-induction and PD-1 re-challenge adds meaningful evidence in a challenging clinical scenario. It highlights the potential for re-treatment even after severe immune-related toxicity, and the importance of individualized risk-benefit assessments. His sustained remission following re-challenge suggests that prior irAEs, do not universally preclude further immunotherapy.

With newer ICIs targeting different pathways coming to clinical practice, strategic deployment of these agents either in combination and/or sequential class rotation will likely become more complex yet beneficial for refractory cases.

## Patient perspective

“I am grateful for all involved with my healthcare. I went about my day as nothing had been wrong”.

## Data Availability

The original contributions presented in the study are included in the article/supplementary material. Further inquiries can be directed to the corresponding author.
